# Integrated Metabolomic and Transcriptomic Analysis Decodes Heat Stress-Induced Metabolic Shifts in Gilt Granulosa Cells

**DOI:** 10.3390/vetsci12111087

**Published:** 2025-11-14

**Authors:** Peng Tang, Xiangyu Si, Xun Xie, Xiaomei Liu, Jianzhen Huang, Yun Shi, Chao Yin

**Affiliations:** College of Animal Science and Technology, Jiangxi Agricultural University, No. 1101 Zhimin Avenue, Nanchang 330045, China; tangpeng@jxnydx1.wecom.work (P.T.); sxy021014@163.com (X.S.); 6020211095@random.com (X.X.); 13576021565@163.com (X.L.); huang813813@jxau.edu.cn (J.H.)

**Keywords:** granulosa cells, metabolic reprogramming, pig, seasonal infertility

## Abstract

While previous studies have extensively demonstrated that summer heat stress (HS) impairs oocyte quality via follicular granulosa cell (GC) mediation, the molecular mechanisms underlying HS-induced GC-mediated oocyte damage—particularly at the multi-omics level—remain poorly understood. This study integrated metabolomic and transcriptomic analyses of GCs from gilts under seasonal thermal stress (winter CON vs. summer HS) to elucidate GC-mediated regulatory networks affecting oocyte quality. Non-targeted metabolomics identified 45 differentially accumulated metabolites (DAMs, *p* < 0.05), with 69% being lipids/lipid-like molecules enriched in pathways such as glycerophospholipid metabolism, choline metabolism, linoleic acid metabolism, the adipocytokine signaling pathway, and the sphingolipid signaling pathway. Parallel transcriptomics revealed 9085 differentially expressed genes (DEGs, *Padj* < 0.05), of which the predominant genes were associated with lipid metabolism, hormone synthesis, and cellular senescence pathways. Cross-omics integration highlighted significant correlations between DAMs and DEGs, particularly for lysoPC(20:4) and 1-hexadecyl-2-eicosatrienoyl-sn-glycero-3-phosphocholine, which showed co-regulation with 69 and 48 genes, respectively. Notably, candidate genes like *TMEM94*, *SLIT3*, *DACT3*, and *CEBPD*, were identified as key regulators of GCs metabolic reprogramming. This study demonstrates for the first time that in vivo HS compromises oocyte developmental competence by disrupting the GC metabolic activities, particularly through lipid metabolism and associated pathways. The identified metabolic signatures and regulatory genes offer mechanistic insights into seasonal infertility and potential biomarkers for thermo-protective strategies in swine reproduction.

## Simple Summary

Employing an integrated multi-omics strategy, this study systematically investigated the metabolic and transcriptional adaptations of porcine granulosa cells (GCs) to seasonal heat stress. GCs were collected from gilt ovaries during both winter control (CON) and summer heat stress (HS) periods, followed by coordinated metabolomic and transcriptomic profiling. The results unveiled profound metabolic reprogramming, with particular emphasis on lipid metabolic pathways, including glycerophospholipid and sphingolipid metabolism. Parallel transcriptomic analysis identified differential expression of pivotal regulatory genes (*TMEM94*, *SLIT3*, *DACT3*, and *CEBPD*) that orchestrate GC metabolic adaptation. These findings pioneer the elucidation of in vivo heat stress-induced dual disruption of metabolic homeostasis and transcriptional networks in GCs, offering novel mechanistic insights into seasonal infertility in gilts. The study not only advances our understanding of thermal stress-induced ovarian dysfunction but also provides potential biomarkers for developing intervention strategies to alleviate seasonal reproductive losses in swine production.

## 1. Introduction

The detrimental impact of summer heat stress (HS) on sow reproduction—manifesting as hormonal imbalances, reduced conception rates, and increased abortions—poses significant challenges to swine production systems [1,2]. This seasonal infertility not only constrains the development of breeding enterprises and regional economies but also leads to a compensatory increase in sow breeding quantity, resulting from the imbalance between pork production and consumer demands, which in turn augments the feeding costs and the difficulty of environmental governance. Previous studies have shown that the success of sow reproduction largely depends on the oocytes, whose quality is determined by two main factors: nuclear and cytoplasmic maturity [3], and the functional status of follicular granulosa cells (GCs) [4]. For example, HS can reduce feed intake and interfere with the activity of the hypothalamus–pituitary–ovarian (HPO) axis, causing nutritional deficiencies and hormonal disorders, and thus leads to the impaired oocyte development and decreased reproductive performance of sows [2,5]. Meanwhile, after entering the antral follicle development stage, the oocyte is located at the center of “cumulus oophorus”; the nutrients and information molecules required for its growth and development are entirely dependent on the provision from the surrounding GCs [4,6]. Therefore, the growth and metabolic status of follicular GCs can directly reflect the developmental level of oocytes. However, compared to oocytes or the studies about GCs in other species, less attention is currently paid to the porcine follicular GCs.

Omics technologies have become important tools in life science research by systematically analyzing the multilevel molecular characteristics of organisms, facilitating the transition from descriptive research to mechanistic understanding in life sciences [7]. However, data from a single omics is insufficient to systematically and comprehensively characterize the molecular regulatory mechanisms of the multifarious biological processes, due to their complexity and globality. For example, transcriptome sequencing (RNA-seq) is devoted to uncovering key genes and regulatory networks, but cannot corroborate the phenotypic changes in cellular activities; similarly, metabolomics is able to detect the end-products during organic biochemical reactions, while it cannot expound the genetic context behind these metabolic alterations [8]. Therefore, multi-omics technology is a more reliable approach that can explore the biological issues from both “cause” and “effect” levels simultaneously, and thus provides in-depth multidimensional insights into the macroscopic developmental processes of organic systems [7]. For example, by integrating transcriptomic and metabolomic analyses, Pan et al. [9] not only characterized the dynamic changes in mRNAs, miRNAs, and metabolites in porcine ovaries across developmental stages and gestation, but also elucidated the regulatory networks between newly identified key genes and metabolic pathways, which may offer new theoretical insights for improving the sow fertility.

Therefore, to elucidate the regulatory networks between key genes and metabolites underlying the metabolic and transcriptional responses of porcine GCs to thermal stress, we conducted an integrated metabolomic and transcriptomic analysis, using GCs collected from gilts during winter (control) and summer (heat stress). Our findings would not only advance the understanding of metabolic and gene expression regulation in sow reproductive physiology but also provide experimental evidence and potential strategies to mitigate the heat stress-induced declines in sow reproductive performance under practical production conditions.

## 2. Materials and Methods

The experimental protocol was approved by the Animal Ethics Committee of Jiangxi Agricultural University (permit No. JXAULL-2020-28). All sampling procedures are in compliance with the “Guidelines on Ethical Treatment of Experimental Animals” (2006) No. 398, set by the Ministry of Science and Technology, China.

### 2.1. Animals and Sample Collection

Cross-bred prepubertal gilts (Landrace × Large White × Duroc) were raised at the experimental farm of Jiangxi Agricultural University (Nanchang, Jiangxi, China) under the uniform indoor conditions, with ad libitum water and diet supply. During winter (early December, CON group) and summer (late August, HS group), gilts aged 135–170 days, weighing 70–120 kg of body weight, and in good health were selected from the herd in four batches (80–100 head per batch) and transported to a local commercial abattoir. Pigs were stunned with an electric shock and then slaughtered, following the Chinese industrial standard.

In each batch, approximately one hundred intact ovaries were dissected from pigs and kept in 0.9% saline (*w*/*v*) supplemented with 75 μg/mL potassium penicillin G and 50 μg/mL streptomycin sulfate at 37 °C, and transported to the laboratory within 2 h. Follicular fluid containing mural granulosa cells and cumulus–oocyte complexes (COCs) was aspirated from 3 to 6 mm follicles in each group with an 18 gauge needle connected to a 10 mL disposable syringe. A total of 80–100 COCs were isolated to evaluate the cumulus expansion and cell viability through the 44 h in vitro maturation (IVM), during which period the COCs were cultured in a 4-well dish with TCM-199 culture medium supplemented with 10% (*v*/*v*) porcine follicular fluid; 10% (*v*/*v*) bovine serum; 15 IU/mL pregnant mare’s serum gonadotropin and human chorionic gonadotropin; 75 μg/mL potassium penicillin G; 50 μg/mL streptomycin sulfate; and 0.8 mmol/L L-glutamine at 38.5 °C, with 5% CO_2_, 20% O_2_, and maximum humidity. Afterwards, the remaining follicular fluid from the same batch of ovaries was pooled together as one sample and centrifuged at 1000 rpm for 5 min, and then the cell precipitate was resuspended in 0.25% trypsin and incubated at 37 °C for 5 min to digest and release the GCs. Finally, the digestion supernatant was filtered through a 200 mesh filter 2–3 times and centrifuged at 1000 rpm for 5 min again to purify the GCs. The cell precipitate was then kept frozen at −80 °C until further analysis.

### 2.2. Evaluation of Cumulus Expansion and Cell Viability

Cumulus expansion was assessed at 24 h during COCs IVM, following the method described previously [10]. Briefly, COCs from each experimental group were retrieved from the incubator and digital images were captured using a charge-coupled device (CCD) camera. The two-dimensional area of each COC was quantified as total pixels using the threshold and measure functions of ImageJ software (version 1.50). Cumulus expansion levels were calculated for each COC as a multiple of difference relative to 0 h IVM. At 44 h of IVM, COCs were digested with hyaluronidase (Hya), and the separated oocytes were evaluated for survival and maturation rates under a light microscope (Leica SAP0, Wetzlar, Germany). The oocytes were defined as having survived if they possessed an intact zona pellucida and plasma membrane, the translucent appearance of cytoplasm, the normal size of the perivitelline space, and an extruded polar body.

### 2.3. Non-Targeted Metabolomics Analysis

LC-MS/MS analyses were performed by Benagen Technology Co., Ltd. (Wuhan, China), using a Vanquish UHPLC system (Thermo Fisher, Wetzlar, Germany). The mobile phase consisted of 25 mmol/L ammonium acetate and ammonia hydroxide in water (pH = 9.75) (A) and acetonitrile (B). The auto-sampler temperature was 4 °C and the injection volume was 2 μL. The Orbitrap Exploris 120 mass spectrometer (Thermo Fisher, Wetzlar, Germany) was used to acquire MS/MS spectra on information-dependent acquisition (IDA) mode in the control of the acquisition software (Xcalibur, version 4.4). The ESI source conditions were set as the following: sheath gas flow rate at 50 Arb, Aux gas flow rate at 15 Arb, capillary temperature at 320 °C, full MS resolution at 60,000, MS/MS resolution at 15,000, collision energy at SNCE 20/30/40, and spray voltage at 3.8 kV (positive) or −3.4 kV (negative), respectively.

The raw data were converted to the mzXML format using ProteoWizard and processed with an in-house program, which was developed using R based on XCMS, for feature detection, extraction, alignment, and integration. The R package (version 4.0) and the BiotreeDB (V3.0) were applied in metabolite identification. The final dataset containing the information of the feature number, sample name, and normalized feature area was imported to the SIMCA software (version 18.0.1) package (Sartorius Stedim Data Analytics AB, Umea, Sweden) for multivariate analysis. Data were scaled and logarithmically transformed to minimize the impact of both noise and high variance of the variables. After these transformations, a 95% confidence interval in the principal component analysis (PCA) score plot was used as the threshold to identify potential outliers in the dataset. Finally, orthogonal projections of latent structures discriminant analysis (OPLS-DA), differentially accumulated metabolites (DAMs) screening, and Kyoto Encyclopedia of Genes and Genomes (KEGG) enrichment were conducted and visualized with R project packages. For differential metabolite screening, the DAMs were filtered by variable importance in projection (VIP) ≥ 1 (from OPLS-DA), and the fold changed upward and downward by 1.5 times and *p* < 0.05.

### 2.4. RNA Sequencing (RNA-seq) Analysis

Total RNA was extracted from GCs portioned from the metabolome samples using the Trizol reagent (Life Technologies, Carlsbad, CA, USA). The K5500^®^Spectrophotometer (Kaiao, Beijing, China) and the Nano 6000 Assay Kit based on the Agilent Bioanalyzer 2100 system (Agilent Technologies, Santa Clara, CA, USA) were used to assess the purity and integrity of total RNA. The mRNAs were separated from the total RNA, interrupted, and reverse-transcribed into the first strand of cDNA using the random hexamer primer and RNase H. The second strand of cDNA was synthesized in the DNA polymerase I system using dNTPs as substrates, and then purified and subjected to end-repair, the addition of poly A tails, and ligation junctions with the VAHTS DNA Clean Beads (Vazyme, Nanjing, China). Afterwards, cDNAs of approximately 250–300 bp were screened for PCR amplification. The constructed library was tested for RNA integrity using the Agilent 2100 bioanalyzer and sequenced on an Illumina X Plus platform at Benagen Technology Co., Ltd. (Wuhan, China).

The raw data were first processed with FastQC (version 0.11.9) to filter out adapters and low-quality sequences. Then, the clean reads were mapped to the *sus scrofa* reference genome (Ensembl_release106), using STAR (version 2.7.9a). RSEM (version 1.3.3) was used to calculate the gene expression level for each sample, expressed as fragments per kilobase of transcript per million fragments mapped (FPKM). The identification of the differentially expressed genes (DEGs) between control and stressed samples was performed by DESeq2 (version 1.34.0), based on the following parameters: fold change ≥ 2.00 and probability ≥ 0.8, with false discovery rate-adjusted *p*-value (FDR) < 0.05. Principal component analysis (PCA) and KEGG enrichment analyses for DEGs were carried out utilizing ClusterProfiler (version 3.8) and R project packages.

### 2.5. Integrated Transcriptome and Metabolome Analysis

Pearson correlation coefficients between identified genes and metabolites in each comparison group are calculated using the cor function from the stats package (version: 4.2.3) in R, based on Pearson’s correlation analysis method. The correlation matrix between DAMs and identified genes is visualized as a heatmap using the pheatmap package (version: 1.0.12) in R. Additionally, DAMs and DEGs with an absolute Pearson correlation coefficient of ≥0.98 are filtered, and a network diagram is plotted using the graph package (Version: 2.0.5) in R.

### 2.6. Quantitative Real-Time PCR (qRT-PCR)

Six DEGs were randomly selected to validate the RNA-seq data through qRT-PCR. Briefly, the total RNA of each sample was extracted and reverse-transcribed into cDNA on an A200 Gradient Thermal Cycler (LongGene, Hangzhou, China), using the SuperScriptTM III First-Strand Synthesis System (Invitrogen, Carlsbad, CA, USA) according to the manufacturer’s protocol. During these operation processes, total RNA was extracted from GCs and its concentration and the purity was quantified with a NanoDrop ND-2000 Spectrophotometer (ThermoFisher, Dover, DE, USA), and then the same quantity (800 ng) of total RNA from each sample was reversely transcribed into cDNA. Subsequently, the cDNA was diluted (1:10, *v*/*v*) and was used for qRT-PCR on a StepOnePlus Real-Time PCR System (Thermo Fisher, Dover, DE, USA), using the TB Green^®^ Premix Ex Taq™ II (TaKaRa, Beijing, China) reaction system. *PPIA* was chosen as a housekeeping gene to normalize the technical variations, and the abundance of mRNA was expressed as the fold change, relative to the mean value of the control group. All primers were synthesized by Tsingke (Beijing, China) (Table S1).

### 2.7. Statistical Analysis

All data are presented as mean ± SEM. For independent samples, t-test and one-way analysis of variance (ANOVA) were applied as a comparison of differences between experimental groups with SPSS 20.0 software (SPSS Inc., Chicago, IL, USA). The method of 2^−ΔΔCt^ was used to analyze the real-time PCR data. The differences were considered statistically significant when *p* < 0.05. The statistical results were plotted on the software GraphPad Prism (version 9.5.1).

## 3. Results

### 3.1. HS Reduced the Cumulus Expansion and Oocyte Maturation

**Figure 1:**
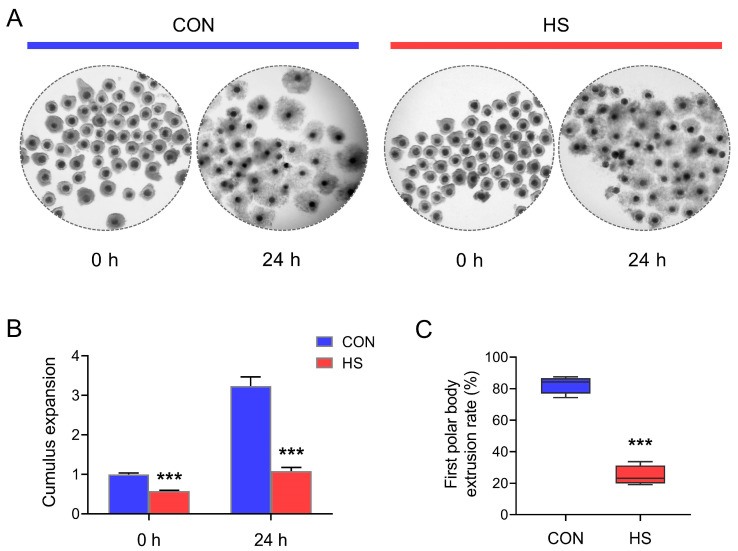
Effect of HS on cumulus expansion and oocyte maturation. (**A**) Representative images (magnification, × 10) of cumulus expansion patterns at different time points during the COCs in vitro maturation; (**B**,**C**) statistical analysis of cumulus expansion and the first polar body extrusion rate of oocytes (*n* = 4). Data are expressed as mean ± SEM. CON: COCs isolated from gilts during winter; HS: COCs isolated from gilts during summer. *** *p* < 0.001, compared with CON group.

As shown in Figure 1, both at 0 h after sampling and 24 h post IVM, the cumulus cell expansion rates of COCs isolated from gilts in summer were significantly (*p* < 0.001) lower than those samples collected in winter (Figure 1A,B). Correspondingly, at 44 h IVM, the first polar body exclusion rate also showed significant (*p* < 0.001) decline in oocytes isolated in summer when compared with those collected in winter (Figure 1C).

### 3.2. HS Altered the Metabolic Profiles of Porcine Follicular Granulosa Cell

**Figure 2:**
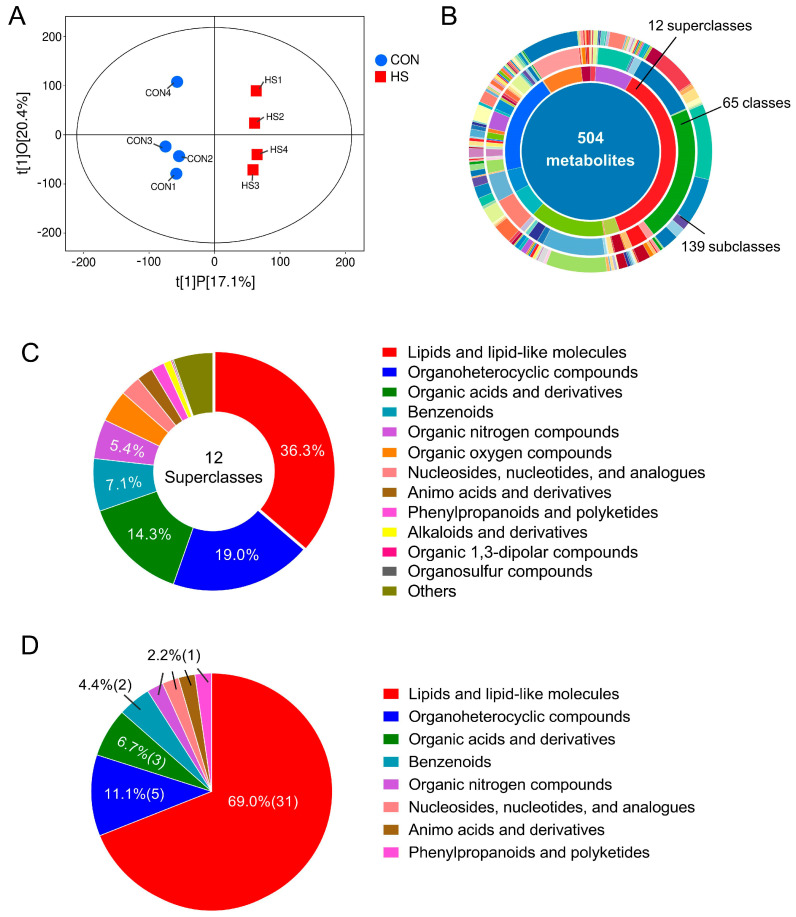
Changes in the metabolic profiles of follicular GCs. (**A**) OPLS-DA analysis of the metabolic profiles between CON and HS groups (*n* = 4); (**B**) number of metabolites identified through LC-MS/MS analysis (*n* = 4); (**C**,**D**) classification and the percentage statistics of total identified metabolites (**C**) and DAMs (**D**) between CON and HS groups (*n* = 4). CON: COCs isolated from gilts during winter; HS: COCs isolated from gilts during summer.

Non-target metabolomics was employed to assess the impact of HS on the metabolome of porcine follicular GCs. OPLS-DA analysis revealed distinct metabolic distributions between CON and HS groups (Figure 2A). LC-MS/MS analysis identified 504 metabolites across 12 superclasses, with lipids/lipid-like molecules being the most abundant (36.3%), followed by organoheterocyclic compounds (19.0%), organic acids and derivatives (14.3%), benzenoids (7.1%), and organic nitrogen compounds (5.4%) (Figure 2B,C). Further analysis identified 45 DAMs, based on variable importance in projection (VIP) ≥ 1 (from OPLS-DA), a fold change threshold of 1.5 (up or down), and statistical significance of *p* < 0.05. Approximately 69% of DAMs belong to the lipids/lipid-like substances, followed by organoheterocyclic compounds (11.1%) and organic acids and derivatives (6.7%) (Figure 2D).

### 3.3. Enrichment and Functional Annotation of DAMs in Response to HS

**Figure 3:**
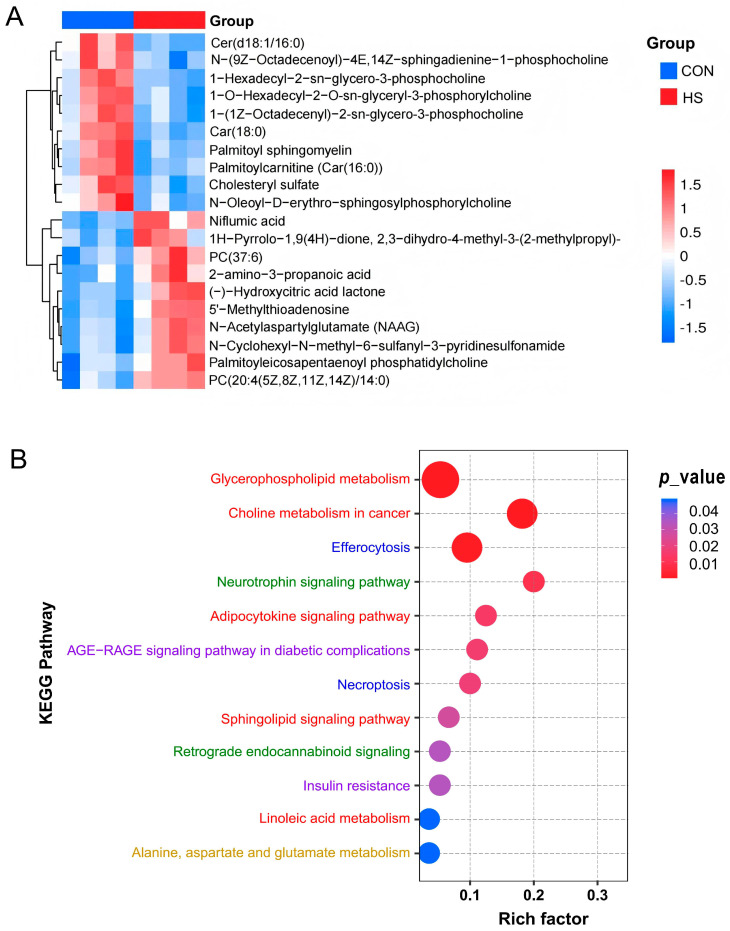
Enrichment analysis of differential metabolites. (**A**) Heatmap of top 10 up- and down-regulated metabolites; (**B**) KEGG pathway enrichment analysis of DAMs. Only DAMs with variable importance in projection (VIP) ≥ 1 (from OPLS-DA), fold change upward and downward by 1.5 times, and *p* < 0.05 were included in this analysis (*n* = 4). The phrases written in different colors represent different types of signal pathways.

As represented in Figure 3A, when comparing with CON group, 14 and 31 DAMs were significantly (*p* < 0.05) up- and down-regulated, respectively, after the high-temperature exposure, including the significantly increased metabolites like niflumic acid, 5′-methylthioadenosine, N-acetylaspartylglutamate, N-cyclohexyl-N-methyl-6-sulfanyl-3-pyridinesulfonamide, (-)-hydroxycitric acid lactone, and propionylcarnitine, etc., as well as the decreased metabolites like 2-cyanoamino-4,6-dihydroxypyrimidine, car(18:0), 2-(hexopyranosyloxy)-3-hydroxypropyl-hexadeca-7,10,13-trienoate, lysoPC(20:4), and 6,7-dihydro-5H-cyclopenta[d]pyrimidine-2,4-diamine, etc. (Table S2).

KEGG pathway enrichment analysis revealed that these DAMs were mainly enriched in the lipid metabolism-related pathways, including the glycerophospholipid metabolism, choline metabolism, linoleic acid metabolism, adipocytokine signaling pathway, and sphingolipid signaling pathway. Secondly, a portion of DAMs were demonstrated to be related to the amino acid metabolism and the metabolic diseases, such as alanine, aspartate and glutamate metabolism, insulin resistance, and the AGE-RAGE signaling pathway, in diabetic complications. In addition, cell transport and death, as well as neural regulation, were also involved in the HS-induced metabolic changes in GCs: for example, cellular efferocytosis, necroptosis, and the neurotrophin signaling pathway (Figure 3B and Table S3).

### 3.4. Differential Expression and Enrichment Analysis of Genes in GCs

**Figure 4:**
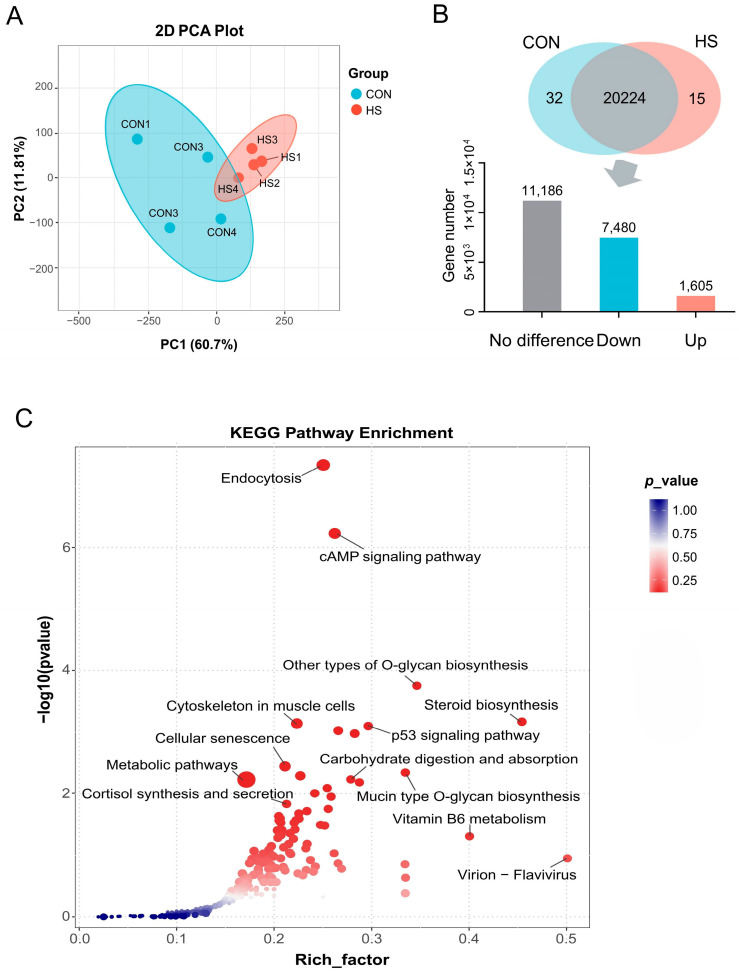
Transcriptional profile changes and enrichment analysis of DEGs between two experimental groups. (**A**) PCA of samples of two experimental groups (*n* = 4); (**B**) regulatory trends of DEGs under HS condition when compared to CON group (*n* = 4); (**C**) KEGG pathway enrichment analysis of DEGs (*n* = 4). CON: COCs isolated from gilts during winter; HS: COCs isolated from gilts during summer.

DEGs were identified and screened by RNA-seq analysis. The PCA plot revealed a clear separation of samples between two experimental groups, indicating the dramatic changes in the gene expression pattern caused by HS (Figure 4A). A total of 9085 DEGs were identified, of which 7480 and 1605 DEGs were significantly (*p* < 0.05) down-regulated and up-regulated in the HS group, respectively, when compared with the CON group (Figure 4B). The accuracy of the RNA-seq data was verified by checking the expression level of six randomly selected DEGs (*TMEM94*, *SLIT3*, *DACT3*, *WDR83*, *CEBPD*, and *ANKS1A*) using the qRT-PCR method, as shown in Figure S1. Afterward, KEGG pathway enrichment analysis was carried out to classify the main approaches through which DEGs regulate the functions of GCs under HS conditions, and the results showed that most of these DEGs were enriched in pathways that were closely tied to the nutrient metabolism, hormone synthesis, and secretion, and cellular activities like endocytosis and senescence (Figure 4C).

### 3.5. Integrated Analysis of Differentially Expressed mRNAs and DAMs

**Figure 5:**
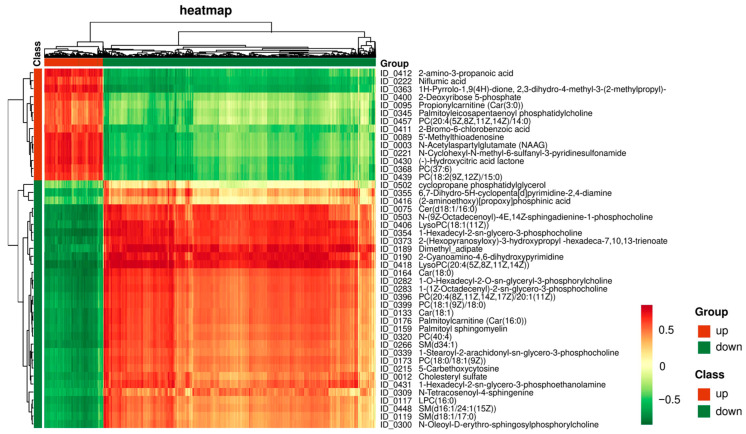
Correlation analysis between DAMs and all identified genes. The representative heatmap shows the regulatory relationships between 45 DAMs and all the identified genes using Pearson’s coefficient. Red (up-regulated) and green (down-regulated) squares indicate the positive and negative regulations of metabolites and genes by HS, separately.

The association analysis between DAMs and the identified genes is shown in Figure 5, using Pearson’s correlation analysis method. The vast majority of genes showed strong correlation with the DAMs, suggesting that the metabolic variations in GCs are regulated by genes referring to extensive biological processes under HS conditions. Subsequently, to obtain a comprehensive insight and explore the regulatory mechanisms between DEGs and DAMs, a network diagram focusing the DAMs and DEGs with an absolute Pearson correlation coefficient of ≥ 0.98 was plotted.

**Figure 6:**
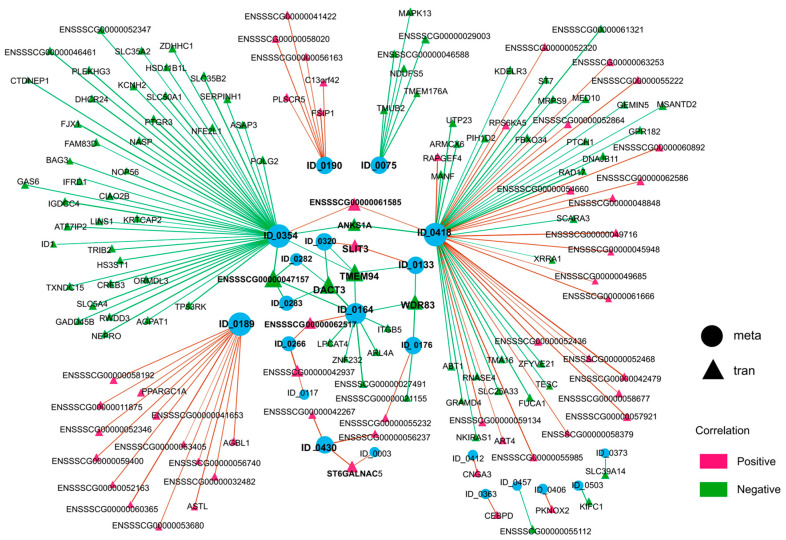
Integrated network analyses of DAMs and DEGs. Correlation analysis between 45 differential metabolites and 9085 DEGs was performed using Pearson’s coefficient. The circle nodes represent metabolites, and the triangle nodes represent transcripts. The blue dots represent the identified metabolites, and the size of dots represents the number of genes that regulate this metabolite. The positive and negative regulatory relationships between DAMs and DEGs are marked by red and green lines and triangles, separately.

As shown in Figure 6 and Table S4, network analysis revealed lysoPC(20:4) (ID_0418) to be the most extensively regulated metabolite (correlated with 69 DEGs), followed by 1-Hexadecyl-2-eicosatrienoyl-sn-glycero-3-phosphocholine (ID_0354, 48 DEGs), dimethyl_adipate (ID_0189, 18 DEGs), car(18:0) (ID_0164, 10 DEGs), cer(d18:1/16:0) (ID_0075, 7 DEGs), 2-Cyanoamino-4,6-dihydroxypyrimidine (ID_0190, 7 DEGs), and (-)-hydroxycitric acid lactone (ID_0430, 3 DEGs), etc. Conversely, from the genetic perspective, multiple genes exhibit significant multifunctionality, being capable of regulating the generation of two or more metabolites. For instance, *TMEM94*, *DACT3* and *ENSSSCG00000047157* are able to regulate four metabolites and *WDR83* can regulate three metabolites, while *ANKS1A*, *SLIT3*, *ST6GALNAC5*, *ENSSSCG00000042937*, *ENSSSCG00000062517*, and *ENSSSCG00000061585* can regulate two. On the contrary, some genes showed highly specific regulatory relationships with DAMs, such as *CEBPD*, *FBXO34*, *ITGB5*, and *DHCR24*.

## 4. Discussion

With the popularization and development of intensive and large-scale farming models during the past decades, reproductive dysfunctions in livestock caused by environmental stressors has become increasingly severe: for example, the declined reproductive performance in sows was induced by summer HS [11,12]. According to the previous studies, oocytes, which act as the ultimate executors of animal reproductive function, were widely favored because of their crucial roles in the mammal reproductive activities [1]. Consistently with these studies, when we conducted in vitro maturation (IVM) culture of COCs, we also found that both the first polar body extrusion rate of oocytes and the expansion rate of cumulus cells were significantly declined in summer when compared with those in early winter.

In the meantime, increasingly, evidence has proven the follicular granulosa cells (GCs, including mural granulosa cells and cumulus cells) to be another critical factor determining the oocyte quality and animal reproductive efficiency under stress conditions, due to their outstanding contributions in providing a stable microenvironment for the oocyte growth [6,13]. Among them, more than 80% of researchers demonstrated that GCs apoptosis and oxidative stress are the primary causes of the decline in oocyte quality [14,15,16], followed by endoplasmic reticulum stress [16,17], mitochondrial dysfunction [18,19], and inflammation [20], whereas the other researchers revealed some new changes or regulatory mechanisms, such as the hormonal metabolic disorders [21,22], abnormal extracellular matrix production [23], and non-coding RNA regulation [24], when they employed the high-throughput detection methods such as transcriptome or metabolome sequencing to obtain global profiles of gene transcription or metabolism in GCs under HS exposure. However, due to the systemic and complex characteristics of cellular activities, data obtained from single-omics investigations and low-throughput detection techniques based on hypothesis-driven experimental designs are insufficient to accurately assess and reveal the mechanisms underlying specific cellular biological processes [8]. Therefore, a combined transcriptomic and metabolomic approach was adopted in this study to explore the harmful effects of HS on porcine GCs. As described in Section 3, a total of 504 reliable metabolites, which belong to 12 subclasses, were identified in our study. Among them, 45 DAMs show significant differences between two experimental groups, and approximately 69% of them were lipids and lipid-like molecules, followed by organoheterocyclic compounds (11.1%) and organic acids and derivatives (6.7%) (Figure 2). KEGG enrichment analysis showed that these DAMs were primarily involved in regulating cellular biological activities such as lipid metabolism, cellular apoptosis and transport, amino acid metabolism, and neural signal conduction. Transcriptome sequencing yielded highly consistent results with metabolomics, of which the identified 9085 DEGs were mainly enriched in pathways related to the substance metabolism (e.g., metabolic pathways, carbohydrate digestion and absorption, vitamin B6 metabolism, O-glycan biosynthesis), hormone synthesis and secretion (e.g., steroid biosynthesis, cortisol synthesis and secretion), cell fate definition (e.g., cellular senescence, p53 signaling pathway), and cytoskeleton organization. Notably, compared with previous studies, our multi-omics approach resolves a critical knowledge gap by demonstrating that lipid metabolic reprogramming—rather than canonical apoptosis/oxidative stress pathways—constitutes the primary response of porcine GCs to in vivo HS. This discrepancy may stem from several factors: first, species differences—most prior studies about GCs focused on large mammals like cattle [20,22] and camels [25] or medical model animals like mice [26]; secondly, differences between in vivo and in vitro experimental conditions—most previous studies used the in vitro HS-treated GCs or COCs as experimental models [23,27], which markedly differ from the GCs directly isolated from gilts in this study; and finally, other factors such as the sampling time points, HS intensity and duration, and etc.

Despite these differences, the rationale for our findings can also be explained by the unique metabolic characteristics of oocytes. Mammalian oocytes are known to exhibit a high lipid droplet content: a physiological trait attributed to their substantial energy demands during meiotic resumption and maturation [18]. Notably, lipids provide a 3.5-fold higher energy yield than glucose, making them the most efficient and convenient energy reserve for oocyte development [28]. However, oocytes lack the capacity for de novo fatty acid synthesis, relying almost entirely on the supply from their surrounding GCs [29]. For instance, when bovine oocytes were cultured without cumulus cells, the number of lipid droplets in the oocytes decreased, and the oocyte development was significantly delayed [30]. Moreover, the critical role of GC lipid metabolism in oocyte growth is further underscored by its regulation of oocyte meiosis and maturation. Numerous lipid metabolites, including cholesterol, lipoproteins, and 14-demethyl-14-dehydrolanosterol, act as steroid hormone precursors and meiotic resumption regulators [31]. Among the 45 DAMs identified in this study, 31 DAMs were significantly reduced under HS compared to the control group, with 24 belonging to the lipids and lipid-like molecules category. This observation makes us reasonably hypothesize that HS may impair oocyte growth and quality by limiting the lipid supply from GCs.

In addition, integrated metabolomic and transcriptomic analysis revealed several candidate genes involved in GC metabolic regulation, which can be categorized into two groups: the “non-specific” regulatory genes (e.g., *TMEM94*, *SLIT3*, *DACT3*, *WDR83*, and *ANKS1A*) which regulate two or more metabolites, and the “specific” genes (e.g., *CEBPD*, *KIFC1*, and *CNGA3*), which regulate only one metabolite (Figure 6). For example, *SLIT3* has been extensively studied for its roles in primordial follicle formation [32] and luteolysis [33] in humans and livestock, as well as in GC proliferation, differentiation, and follicle selection via the *SLIT/ROBO* pathway during hens’ prehierarchical follicle development [34,35]. Similarly, *CEBPD*, a member of the CCAAT/enhancer binding protein (CEBP) family, was reported to function as a transcription factor involved in the cell death initiation [36] and hormone production [37] in mammalian mammary glands and ovaries. Despite the current lack of reports linking these genes to livestock reproduction, their identification presents a crucial opportunity for future research to decode the regulatory mechanisms underlying animal reproduction.

## 5. Conclusions

Through integrated metabolomic and transcriptomic analyses, we identified that lipid metabolism and its associated signaling pathways drive the primary cellular changes in porcine GCs under summer HS. Notably, several novel candidate genes, such as *TMEM94*, *SLIT3*, *DACT3*, and *CEBPD*, play crucial roles in regulating this process. These findings not only elucidate the mechanisms underlying HS-induced GC dysfunction but also highlight potential therapeutic targets for mitigating summer heat stress-related reproductive impairments in livestock.

## Data Availability

The raw data supporting the conclusions of this article will be made available by the authors on request.

## Supplementary Materials

The following supporting information can be downloaded at: https://www.mdpi.com/article/10.3390/vetsci12111087/s1. Table S1: Primer sequences of qRT-PCR; Table S2: The list of differential accumulated metabolites; Table S3: KEGG pathway enrichment of differential accumulated metabolites; Table S4: Correlation network analysis of DAMs and DEGs; Figure S1: Verification of differential expressed genes identified through RNA-seq. The expression level of six candidate genes including *TMEM94*, *SLIT3*, *DACT3*, *WDR83*, *CEBPD*, and *ANKS1A* were detected to verify the accuracy of RNA-sequencing (*n* = 4). Data are expressed as mean ± SEM. CON: COCs isolated from gilts during winter; HS: COCs isolated from gilts during summer. * *p* < 0.05, ** *p* < 0.01, and *** *p* < 0.001, compared with CON group;
