# Supplementary material for: Integrated Metabolomic and Transcriptomic Analysis Decodes Heat Stress-Induced Metabolic Shifts in Gilt Granulosa Cells
Source: Vet Sci. 2025 Nov 14;12(11):1087. doi: 10.3390/vetsci12111087 (PMC12656734; doi:10.3390/vetsci12111087)
Supplement: Supplementary file 1 [file vetsci-12-01087-s001.zip › Table S1.pdf]

Table S1 Primer sequences for qRT-PCR

Target genes	Sequences (5’→3’)	Products, bp	GenBank No.
TMEM94	F：CAGGCAGAGCTATGGAAGGC<br>R：GCTCCTTCAGGTGACACTCC	139	XM_021066634.1
SLIT3	F：CTTGGCTCTGGCGAGTGTT<br>R：TGAGTCCAGCGAAATCCGTC	197	XM_021076953.1
DACT3	F：CACCTTCGACCTTCTGTGGG<br>R：GCGTCTCCTAGAGACTTGGG	118	XM_003355955.4
WDR83	F：AGTACAAGCTGGACTGCTGC<br>R：GTCAGCTTCATAGGCCTCTTCC	227	XM_003123321.6
CEBPD	F：GCGCTCTTCAGCCTAGACG<br>R：CTCTCGTCGTCGTACATGGC	137	XM_005663091.2
ANKS1A	F：CTTCCGGATCCCAGAAGAGC<br>R：CTGCAGGTCCTTGGTGATGT	317	NM_001246264.2
PPIA	F：GACTGAGTGGTTGGATGG<br>R：TGATCTTCTTGCTGGTCTT	116	NM_214353.1
