# Supplementary figures and images for: Integrated Metabolomic and Transcriptomic Analysis Decodes Heat Stress-Induced Metabolic Shifts in Gilt Granulosa Cells
Source: Vet Sci. 2025 Nov 14;12(11):1087. doi: 10.3390/vetsci12111087 (PMC12656734; doi:10.3390/vetsci12111087)

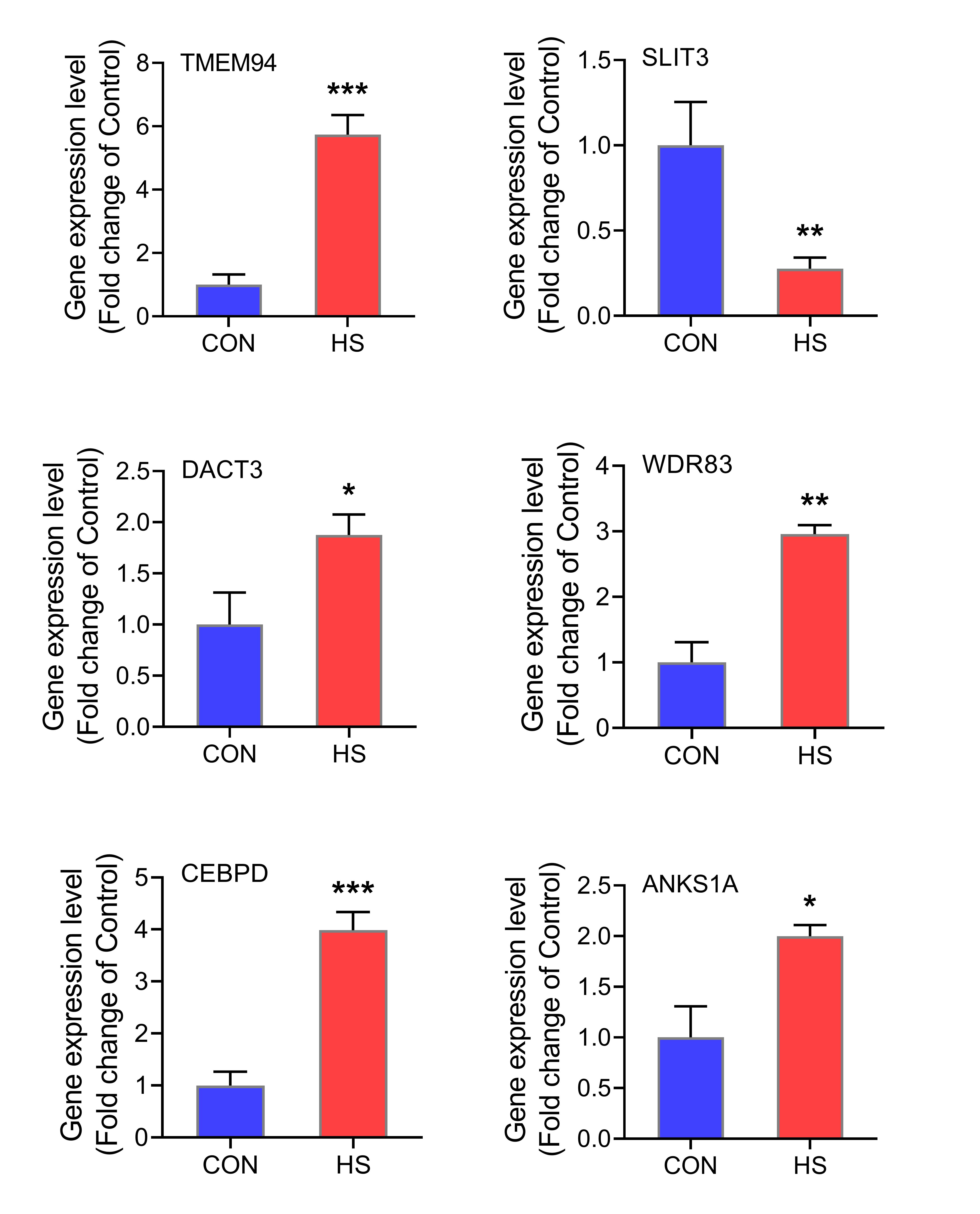

Supplement: Supplementary file 1 [file vetsci-12-01087-s001.zip › Figure S1.jpg]
